# Identification of Neutrophil Activation Markers as Novel Surrogate Markers of CF Lung Disease

**DOI:** 10.1371/journal.pone.0115847

**Published:** 2014-12-29

**Authors:** Timo Rath, Lisa Zwaschka, Lisa Hage, Marion Kügler, Katrin Menendez, Lutz Naehrlich, Richard Schulz, Martin Roderfeld, Elke Roeb

**Affiliations:** 1 Justus-Liebig-University Giessen, Department of Gastroenterology, Giessen, Germany; 2 Friedrich Alexander University Erlangen, Department of Medicine 1, Erlangen, Germany; 3 Justus-Liebig-University Giessen, Department of Pediatrics, Division of Pulmonology, Giessen, Germany; 4 Justus-Liebig-University Giessen, Department of Pulmonology, Giessen, Germany; INRS, Canada

## Abstract

**Background and aims:**

Cystic Fibrosis (CF) lung disease is characterized by progressively declining lung function and represents a major factor contributing to the high morbidity and mortality associated with CF. However, apart from spirometry, respiratory disease surrogate markers reliably indicating CF lung disease and the occurrence of pulmonary exacerbations (PEx) are still lacking. Within this study, we aimed to identify new experimental biomarkers for the detection of CF lung disease.

**Methods:**

54 adult and 26 pediatric CF patients were included in the study and serum concentrations of MMP-1, -2, -8, -9, -13, TIMP-1, TIMP-2, YKL-40, hyaluronic acid, procollagen III peptide were quantified by ELISA. CF lung disease was diagnosed by lung function test, PEx was defined based on a clinical scoring established by Rosenfeld in 2001.

**Results:**

Adults and children with moderate to severe CF lung disease exhibited significantly increased serum expression of MMP-8, MMP-9, YKL-40 and TIMP-1. Further, MMP-8, MMP-9 and YKL-40 were significantly increased in adult CF patients suffering from PEx compared to those without clinical signs of respiratory exacerbation. MMP-8, MMP-9, YKL-40, and TIMP-1 serum levels were unaffected by the presence or absence of CF liver disease or pancreatic insufficiency.

**Conclusions:**

MMP-8, MMP-9, and YKL-40 might serve as novel non-invasive biomarkers of CF lung disease and PEx.

## Introduction

Cystic fibrosis (CF) is the most common autosomal recessive inherited disease among caucasians with a highly variable genotype and phenotype. Although generally affecting many organs, lung disease accounts for the majority of increased morbidity and mortality associated with CF [1]. Clinically, CF lung disease is characterized by mucostasis, mucus hypersecretion and nonresolving neutrophilic inflammation. Worsening of respiratory symptoms such as increased cough or sputum production, or systemic symptoms such as weight loss, anorexia or fatigue episodically occur in CF patients and are commonly referred to as pulmonary exacerbations (PEx) [2]. Despite the critical role that both, progressively declining lung function in CF patients and recurrent episodes of PEx play in daily clinical routine, no other non-invasive surrogate markers for existence and progression of CF lung disease apart from spirometry have been validated or are in wide clinical use. Especially for PEx, neither a standardized definition nor a reliable laboratory marker defining PEx has entered clinical routine. Therefore, the diagnosis of PEx is essentially based on clinical evaluation, and there are considerable variations in identifying and treating PEx, both between centers and within the same center [3], [4]. Given these considerations it becomes apparent that a routine marker reliably indicating the existence and/or worsening of CF lung function and the occurrence of PEx is of clinical need.

Pathophysiologically, CF lung disease is characterized by an abnormal tissue remodeling and the net accumulation of extracellular matrix (ECM), leading to subsequent scar formation and ultimately bronchial fibrosis. Mainly from intensive research on liver and lung fibrosis, a growing understanding of the pathogenesis of fibrogenesis identified non-invasive quantitative serum fibrosis markers, which are pathophysiologically derived from extracellular matrix (ECM) turnover and might directly translate the molecular pathogenesis of fibrogenesis into clinical application [5], [6], [7], [8]. Further, several of these matrix markers are also released by neutrophils or induce neutrophil chemokinesis [9], [10], [11], [12]. Based on these considerations we hypothesized that matrix or neutrophil markers might be associated with impaired lung function and the occurrence of PEx. Within this study we quantified a large panel of experimental matrix and neutrophil markers in the serum of CF patients and addressed the questions whether these might serve as biomarkers or surrogate markers of CF lung disease and PEx.

## Materials and Methods

### Patients

This study has been conducted according to the principles expressed in the Declaration of Helsinki. Written informed consent was obtained from all participating patients or their parents. The study was approved by the ethics committee of the medical faculty of the Justus-Liebig-University Giessen (Klinikstraße 36, 35392 Giessen, Germany) with the approval no. 75/09. The diagnosis of CF was established by sweat test and later confirmed by genetic tests in all subjects. All patients were treated according to European and U.S. guidelines [13], [14]. Patients were seen in the CF outpatient clinic every 3 months. At each visit the following parameters were routinely assessed: patient history and physical examination, sputum and blood samples, and lung function tests including blood gas analysis (BGA). Serum samples were taken during the office hours of the CF outpatient clinic (from 8 AM to 2 PM) for the determination of CRP, leukocytes and liver enzymes. In case of PEx, serum samples were taken before the admission of antibiotics. Pulmonary function tests were carried out using body plethysmography according to established guidelines [15] on a MasterScreen V4.6 (VIASYS Healthcare GmbH, Hoechberg, Germany). All values were normalized to weight adapted normal values with respect to the individual body-mass-index (BMI). To address whether matrix and neutrophil markers can serve as biomarkers of CF lung disease, we assessed their serum expression in CF patients with a forced expiratory volume in one second (FEV1) below and above 80%, with a vital capacity (VC) of below and above 80%, and with a ratio between FEV1 and VC below and above 70% (FEV1/VC), all of which serve as established indicators of CF lung disease and have been the primary outcome in many clinical trials [13], [16], [17], [18]. For the purpose of statistical analysis, the following terminology of CF lung disease was utilized: (i) patients with a decline of FEV1 and VC below 80% and a FEV1/VC below 70%: moderate to severe CF lung disease, (ii) patients with a FEV1 and VC≥80% and a FEV1/VC ratio ≥70%: mild CF lung disease. Patients were instructed to report to the clinic immediately whenever symptoms suggestive of acute pulmonary exacerbation (PEx) appeared. PEx was defined based on the PEx score established by Rosenfeld in 2001 [19]. The PEx score consists of six common clinical findings each weighted by a certain coefficient (in brackets): decreased exercise tolerance (1.8), increased cough (1.5), increased cough/sputum congestion (1.5), school or work absenteeism (1.6), increased adventitial sound on lung examination (1.2), and decreased appetite (1.1) [19]. Pulmonary exacerbation was presumed when the PEx score exceeded the critical value of 2.6. If senior CF specialists considered the exacerbation severe enough systemic antibiotic therapy was instituted. Choice of antibiotic therapy was based on the results of the most recent sputum culture. Cystic fibrosis liver disease (CFLD) was diagnosed according to recent guidelines [20] if at least two of the following conditions on at least two consecutive examinations spanning a one-year period were present: (i) Hepatomegaly (liver span>2 cm below the costal margin on the medioclavicular line) confirmed by ultrasound, (ii) two abnormal serum liver enzyme levels (ALT, AST, γGT> ULN), (iii) ultrasound abnormalities other than hepatomegaly (increased, heterogeneous echogenicity, nodularity, irregular margins).

### Routine laboratory tests and determination of experimental matrix and neutrophil markers

All patients underwent routine haematological and biochemical investigations on the day of quantification of serum biomarkers. The following arrays and assays were used to quantify serum levels of experimental biomarkers: Serum MMP-1, -2, -8, -9, and -13 protein levels were measured using the Fluorokine MAP multiplex human MMP panel (R&D Systems, Minneapolis, USA). TIMP-1, TIMP-2, C-terminal procollagen III peptide (PIIIP), hyaluronic acid (HA) and YKL-40 were determined using commercially available kits with a serum dilution of 1∶200 for TIMP-1 and TIMP-2, 1∶13 for PIIIP, 1∶1 for HA and undiluted for YKL-40 (MMP-9: Cat.No. DY911; TIMP-1: Cat.No. DY970; TIMP-2: Cat.No. DY971, all R&D Systems, Weinheim, Germany; PIIIP: Cat.No. E3087Hu, YKL-40: Cat.No. 8020, both from Quidel, San Diego, USA; HA: Cat.No. TE1017, Osteomedical, Bünde, Germany).

### Statistical analysis

Statistical analysis was performed with SPSS 21.0 (SPSS Inc, Chicago, Ill). Normal distribution of the data was tested using the Kolmogorov–Smirnov test and visualization of histograms. Failing to meet criteria for normal distribution, were analyzed using non-parametric tests. Expression of serum markers is shown in Box-and-Whisker Plots. The upper and lower hinges of the box represent the 75^th^ and 25^th^ percentile, respectively. The line indicates the median value; error bars represent the minimum and maximum. Values deviating from the box by 1.5- to 3-fold interquartile range were defined as outliers (o). Significant differences are pointed out (*p<0.05, **p<0.01).

## Results

### Patient characteristics

Clinical and demographic data of the 54 adult and 26 pediatric CF patients that were included in the study are presented in **Table 1** **and** **2**, respectively.

**Table 1:** Demographic and clinical data of the adult CF patient cohort.

	Adult CF patients (n = 54)		
	Mild CF lung disease(n = 34)	Moderate to severe CF lungdisease (n = 20)	Significance
**Demographic and clinical data**			
Men (n)	21	10	p = 0.403
Women (n)	13	10	
Age (y)			
mean (median) ± SD	32.2 (31)±8.8	30.9 (30) ±7	p = 0.647
BMI (kg/m^2^)			
mean (median) ± SD	21.2 (21)±2.5	20.2 (20) ±2.7	p = 0.119
CFLD (n)			
no CFLD	16	6	p = 0.222
CFLD	18	14	
%FEV1			
mean (median) ± SD	73 (73) ±19	40 (39) ±13	p<0.001
%VC			
mean (median) ± SD	86 (88) ±16	69 (73) ±17	p = 0.003
**Biochemistry**			
Hemoglobin (g/dL)			
mean (median) ± SD	14.1 (14.4) ±2.1	14.2 (14.5) ±1.7	p = 0.971
range	8.5–17.1	10.5–16.9	
Platelet count (G/L)			
mean (median) ± SD	282 (282) ±103	295 (282) ±99	p = 0.837
range	13−514	113−541	
Alanine aminotransferase (U/L			
mean (median) ± SD	27 (25) ±16	24 (21) ±12	p = 0.622
range	7−83	10−55	
γ-glutamyl transpeptidase (U/L)			
mean (median) ± SD	32 (19) ±31	35 (19) ±38	p = 0.762
range	9−114	6−137	
Bilirubin (mg/dL)			
mean (median) ± SD	1.4 (0.9) ±1.9	0.7 (0.6) ±0.5	p = 0.511
range	0.1–7	0.2–1.8	
Albumin (g/dL)			
mean (median) ± SD	4.3 (4.4) ±0.38	4.2 (4.2) ±0.39	p = 0.437
range	3.4–4.9	3.3–4.6	
Prothrombin time (%)			
Mean (median) ± SD	89 (97) ±22	91 (90) ±13	p = 0.625
range	21−112	72−116	
CrP (G/L)			
mean (median) ± SD	17 (7) ±48	20 (10) ±31	p = 0.163
range	0−285	0−135	

**Table 2:** Demographic and clinical data of the pediatric CF patient cohort.

	Pediatric CF patients (n = 26)		
	Mild CF lungdisease (n = 19)	Moderate to severe CFlung disease (n = 7)	Significance
**Demographic and clinical data**			
Men (n)	7	6	p = 0.063
Women (n)	12	1	
Age (y)			
mean (median) ± SD	11.5 (13)±5.5	15 (16) ±2.9	p = 0.025
BMI (kg/m^2^)			
mean (median) ± SD	−0.22 (−0.5) ±1.1	−1.34 (−1.7) ±1.1	p = 0.041
CFLD (n)			
no CFLD	11	5	p = 0.611
CFLD	8	2	
%FEV1			
mean (median) ± SD	91 (94) ±15	61 (65) ±23	p = 0.004
%VC			
mean (median) ± SD	87 (89) ±19	79 (76) ±29	p = 0.657
**Biochemistry**			
Hemoglobin (g/dL)			
mean (median) ± SD	13.8 (13.8) ±0.9	14.2 (14.2) ±1.2	p = 0.364
range	12.4–15.6	12.5–16	
Platelet count (G/L)			
mean (median) ± SD	329 (326) ±64	346 (332) ±80	p = 0.651
range	217−418	217−484	
Alanine aminotransferase (U/L			
mean (median) ± SD	36 (30) ±19	26 (24) ±15	p = 0.156
range	17−85	12−54	
γ-glutamyl transpeptidase (U/L)			
mean (median) ± SD	22 (16) ±24	39 (18) ±45	p = 0.231
range	7−115	13−138	
Bilirubin (mg/dL)			
mean (median) ± SD	0.5 (0.3) ±0.4	0.3 (0.3) ±0.1	p = 0.971
range	0.2–1.8	0.1–0.4	
Albumin (g/dL)			
mean (median) ± SD	4.4 (4.4) ±0.2	4.4 (4.5) ±0.4	p = 0.974
range	4.1–4.7	3.9–4.8	
Prothrombin time (%)			
Mean (median) ± SD	87 (90) ±11	86 (84) ±7	p = 0.828
range	68–106	76–95	
CrP (G/L)			
mean (median) ± SD	1 (0) ±3	8 (8) ±6	p = 0.004
range	0−12	0−17	

***BMI SDS*** *, Body Mass Index Standard Deviations Score.*

### Lung function and biomarker expression

**Figure 1:**
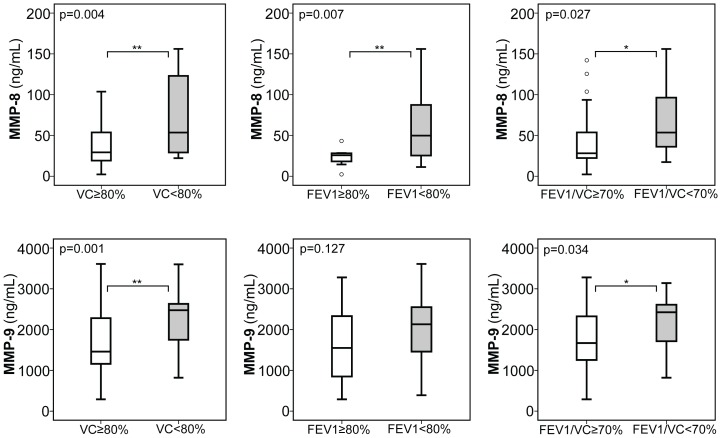
Serum expression of MMP-8 and MMP-9 in adult CF patients with CF lung disease. In adult CF patients serum MMP-8 levels were consistently increased in patients with moderate to severe CF lung disease, as assessed by declined %FEV1 and %VC below 80% and a declined ratio of FEV1/VC below 70%, compared to those with mild CF lung disease (FEV1&VC: ≥80%; FEV1/VC ratio: ≥70%). Serum MMP-9 was also significantly increased in CF patients with decreased %VC and decreased FEV1/VC. Upper and lower hinge: 75th and 25th percentile, respectively; Line: median value; Error bars: minimum and maximum (*p<0.05, **p<0.01).

When stratifying our adult patient cohort according to previously defined established parameters of CF lung disease [13], [16], [17], [18], a total of 20 adults with CF exhibited a decline of FEV1 and VC below 80% and a FEV1/VC ratio below 70%, all indicative of moderate to severe CF lung disease, and we observed a robust and significant increase of MMP-8 and YKL-40 in these patients with moderate to severe CF lung disease compared to those without a relevant decline in lung function or only mild CF lung disease (as indicated by a FEV1 and VC above 80% or a FEV1/VC ratio above 70%) (**Figs. 1** **and** **2**). Further, serum expression of MMP-9 and TIMP-1 were significantly increased in CF patients with a declined VC (MMP-9 and TIMP-1) and FEV1 (TIMP-1) or a declined FEV1/VC ratio (MMP-9) (**Figs. 1** **and** **2**). In contrast, serum expression of MMP-1, -2, -13, TIMP-2, hyaluronic acid (HA), and procollagen III peptide (PIIIP) was unchanged between CF patients with a FEV1 (**S1 Table**) or VC (**S2 Table**) below and above 80%, or a ratio of FEV1/VC (**S3** **Table**) above and below 70%.

**Figure 2:**
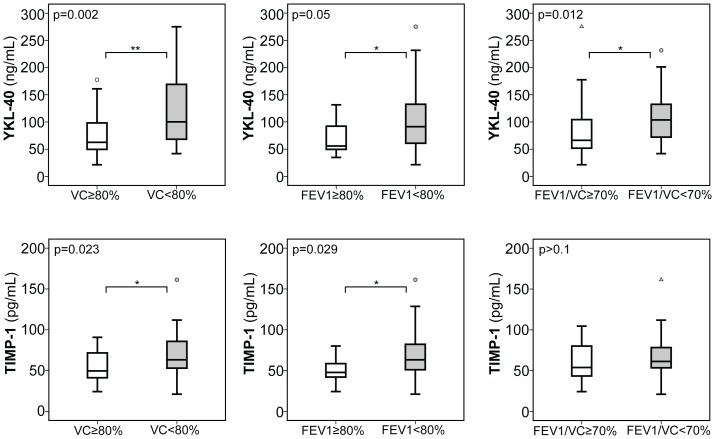
Serum expression of YKL-40 and TIMP-1 in adult CF patients with CF lung disease. In adult CF patients serum YKL-40 levels were consistently increased in patients with moderate to severe CF lung disease, as assessed by declined %FEV1 and %VC below 80% and a declined ratio of FEV1/VC below 70%, compared to those with mild CF lung disease (FEV1&VC: ≥80%; FEV1/VC ratio: ≥70%). Serum TIMP-1 was also significantly increased in adult CF patients with decreased %VC and decreased %FEV1. Upper and lower hinge: 75th and 25th percentile, respectively; Line: median value; Error bars: minimum and maximum. (*p<0.05, **p<0.01).

Liver and pancreas represent two other organ systems that are, apart from the lung, frequently affected by CF and thus might act as potential confounders of the observed up-regulation of MMP-8, MMP-9, YKL-40 and TIMP-1 in CF lung disease. Hence, we also assessed biomarker serum expression in patients with and without CF associated liver disease (CFLD) as diagnosed according to recent established guidelines [20], Importantly, none of the biomarkers differed between CF patients with and without CFLD (**Table 3**). Regarding pancreatic insufficiency, only 3 patients of the adult CF cohort had no pancreatic insufficiency, while 51 patients were pancreatic insufficient. In these exploratory analyses, none of the biomarkers exhibited significant differences between adults with and without pancreatic insufficiency either. Further, CF patients stratified according to lung function into those with mild or moderate to severe CF lung disease did not exhibit any differences in laboratory or clinical markers indicative of CF liver disease (**Table 1**). Together, these data indicate that the observed increased expression of MMP-8, MMP-9, YKL-40, and TIMP-1 occur indeed relatively specific for the existence of CF lung disease without being affected by pancreas and liver disease as other major manifestations of CF.

**Table 3:** Serum expression of matrix and neutrophil markers in adult CF patients without (n  = 22) and with (n  = 32) CFLD.

	without CFLD	with CFLD	*Significance*
**YKL-40** (ng/mL)			
Mean ± SD	85.2±38.5	105.2±62.8	p = 0.360
Median (range)	83.6 (31.2–172.8)	81.3 (21.4–275)	
**MMP-1** (ng/mL)			
Mean ± SD	1593±925	1446±795	p = 0.660
Median (range)	1430 (450–4160)	1285 (410–3430)	
**MMP-2** (ng/mL)			
Mean ± SD	12.44±2.6	13.09±2.4	p = 0.379
Median (range)	12.76 (6.7–18.6)	13.14 (8.02–18.45)	
**MMP-8** (ng/mL)			
Mean ± SD	54.9±45.2	59.7±43.3	p = 0.771
Median (range)	43.3 (11.3–178.1)	49.2 (2.3–156.1)	
**MMP-9** (ng/mL)			
Mean ± SD	1896±692	2057±877	p = 0.400
Median (range)	1690 (85–3280)	2220 (290–3610)	
**MMP-13** (ng/mL)			
Mean ± SD	19.27±15.34	49.26±151.26	p = 0.298
Median (range)	11.93 (1.6–41)	4.96 (0–651)	
**TIMP-1**(ng/mL)			
Mean ± SD	59.3±30.4	66.9±23.1	p = 0.149
Median (range)	53.5 (21.1–161.3)	63.2 (27.9–128.7)	
**TIMP-2** (pg/mL)			
Mean ± SD	123.33±18.17	129.94±30.1	p = 0.606
Median (range)	120.72 (89.2–149)	125.7 (80.8–211.1)	
**HA** (ng/mL)			
Mean ± SD	22.35±15.36	32.72±25.96	p = 0.143
Median (range)	21.99 (0.79–50.3)	28.96 (7.2–127.5)	
**PIIIP** (ng/mL)			
Mean ± SD	14.85±29.45	14.32±23.6	p = 0.898
Median (range)	5.02 (1.2–136.1)	4.88 (0–122)	

**Figure 3:**
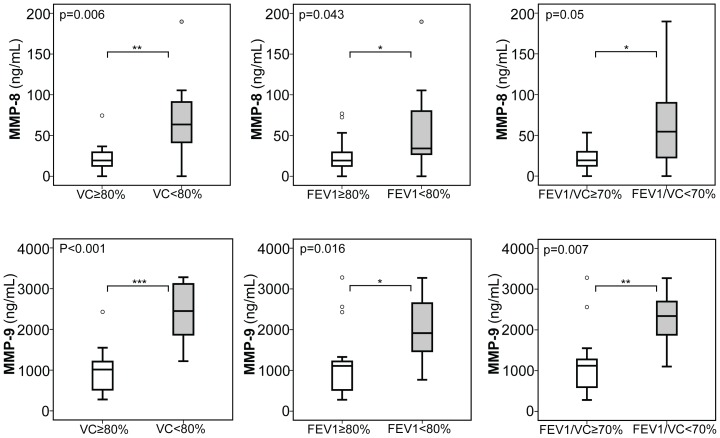
Serum expression of MMP-8 and MMP-9 in pediatric CF patients with CF lung disease. Serum levels of both, MMP-8 and MMP-9 in children with moderate to severe CF lung disease, as assessed by declined %FEV1 and %VC below 80% and a declined ratio of FEV1/VC below 70%, compared to children with mild CF lung disease (FEV1&VC: ≥80%; FEV1/VC ratio: ≥70%). Upper and lower hinge: 75th and 25th percentile, respectively; Line: median value; Error bars: minimum and maximum. (*p<0.05, **p<0.01).

**Figure 4:**
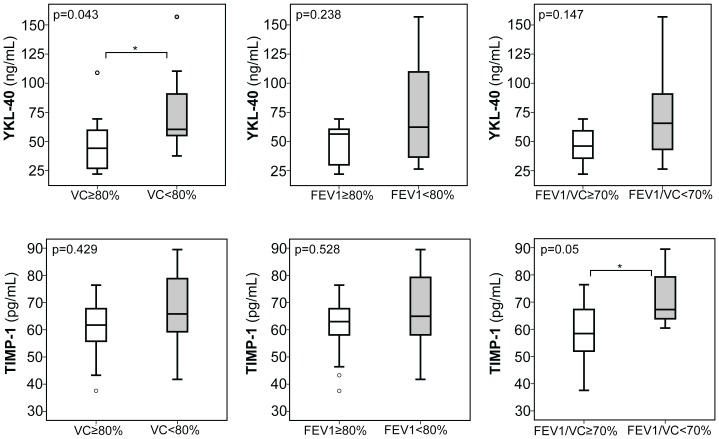
Serum expression of YKL-40 and TIMP-1 in pediatric CF patients with CF lung disease. Serum levels of YKL-40 were significantly increased in children with reduced %VC and increased by trend in children with a reduced FEV1/VC ratio. TIMP-1 was increased in CF children with a reduced FEV1/VC ratio below 70% compared to those with a ratio above 70%. Upper and lower hinge: 75th and 25th percentile, respectively; Line: median value; Error bars: minimum and maximum. (*p<0.05, **p<0.01).

To further substantiate the association of the aforementioned biomarkers with CF lung disease, we turned our attention to a cohort of 26 pediatric CF patients, of which clinical and demographic data are summarized in **Table 2**. In these CF children, we also assessed serum expression of the whole panel of ECM markers and similarly to our observations in adult CF patients, when CF children were stratified according to FEV1 and VC below or above 80% or a ratio of FEV1/VC below or above 70%, we found significantly increased serum levels of MMP-8 and MMP-9 (**Fig. 3**) with each of these stratification indicative of moderate to severe CF lung disease. Further, YKL-40, and TIMP-1 were significantly increased in CF children with reduced VC (YKL-40) or FEV/VC ratio (TIMP-1) (**Fig. 4**). Similar to our observations in adults, serum expression of MMP-1, -2, -13, TIMP-2, HA, and PIIIP was unchanged between pediatric CF patients with a FEV1 (**S4** **Table**) or VC (**S5 Table**) below and above 80%, or a ratio of FEV1/VC (**S6 Table**) above and below 70%.

Similar to the analyses performed in adults, we then analyzed biomarker expression in CF children in those with and without CFLD and analyzed biomarker expression in these subgroups. Consistent with our observations in adults, CF children with lung disease exhibited a significant decrease in parameters of pulmonary function and a slight increase in CrP levels whereas other laboratory or clinical markers were unchanged (**Table 2**) between children with and without CF lung disease. Importantly, serum biomarker expression was unaltered in CF children with and without CFLD (**Table 4**). Regarding pancreatic insufficiency, only 2 children with CF were pancreatic sufficient, thereby only allowing exploratory analyses. However, in these exploratory analyses, none of the analyzed biomarkers differed between CF children with and without pancreatic insufficiency. Thus these results indicate that serum expression of MMP-8, MMP-9, YKL-40, and TIMP-1 are also associated with declined lung function in CF children and that this association is indeed rather specific to lung disease and not affected by CFLD or pancreatic insufficiency, although the latter analysis has only exploratory character.

**Table 4:** Serum expression of matrix and neutrophil markers in pediatric CF patients without (n  = 16) and with (n  = 10) CFLD.

	without CFLD	with CFLD	*Significance*
**YKL-40** (ng/mL)			
Mean ± SD	52.8±23.2	69.1±40.8	p = 0.412
Median (range)	50.4 (22.1–114.6)	59.2 (22–157)	
**MMP-1** (ng/mL)			
Mean ± SD	1074±787	1381±992	p = 0.521
Median (range)	800 (350–3460)	1270 (240–3430)	
**MMP-2** (ng/mL)			
Mean ± SD	17.6±3.8	19.7±4.97	p = 0.238
Median (range)	16.9 (12.6–26.8)	19.7 (12.91–28.34)	
**MMP-8** (ng/mL)			
Mean ± SD	45.4±46.2	21.6±15.5	p = 0.159
Median (range)	27.1 (0–189.7)	19.3 (0–54.6)	
**MMP-9** (ng/mL)			
Mean ± SD	1537±863	1201±879	p = 0.204
Median (range)	1275 (280–3280)	830 (340–3270)	
**MMP-13** (ng/mL)			
Mean ± SD	62.59±58.4	71.8±40.4	p = 0.317
Median (range)	49.3 (0–249)	70.7 (0.2–131.7)	
**TIMP-1**(ng/mL)			
Mean ± SD	61.2±8.8	61.1±19.8	p = 1.00
Median (range)	61.7 (41.8–72.9)	58.4 (27.6–89.5)	
**TIMP-2** (pg/mL)			
Mean ± SD	131.4±25.3	150.4±23.2	p = 0.084
Median (range)	133.5 (94.7–178)	149 (125.6–210)	
**HA** (ng/mL)			
Mean ± SD	19.2±10.4	22.9±16.3	p = 0.642
Median (range)	17.5 (0–40.3)	26.6 (0–45.1)	
**PIIIP** (ng/mL)			
Mean ± SD	9.1±12.2	21±39.1	p = 0.707
Median (range)	4.3 (2–50.5)	8.2 (0–136.1)	

**Figure 5:**
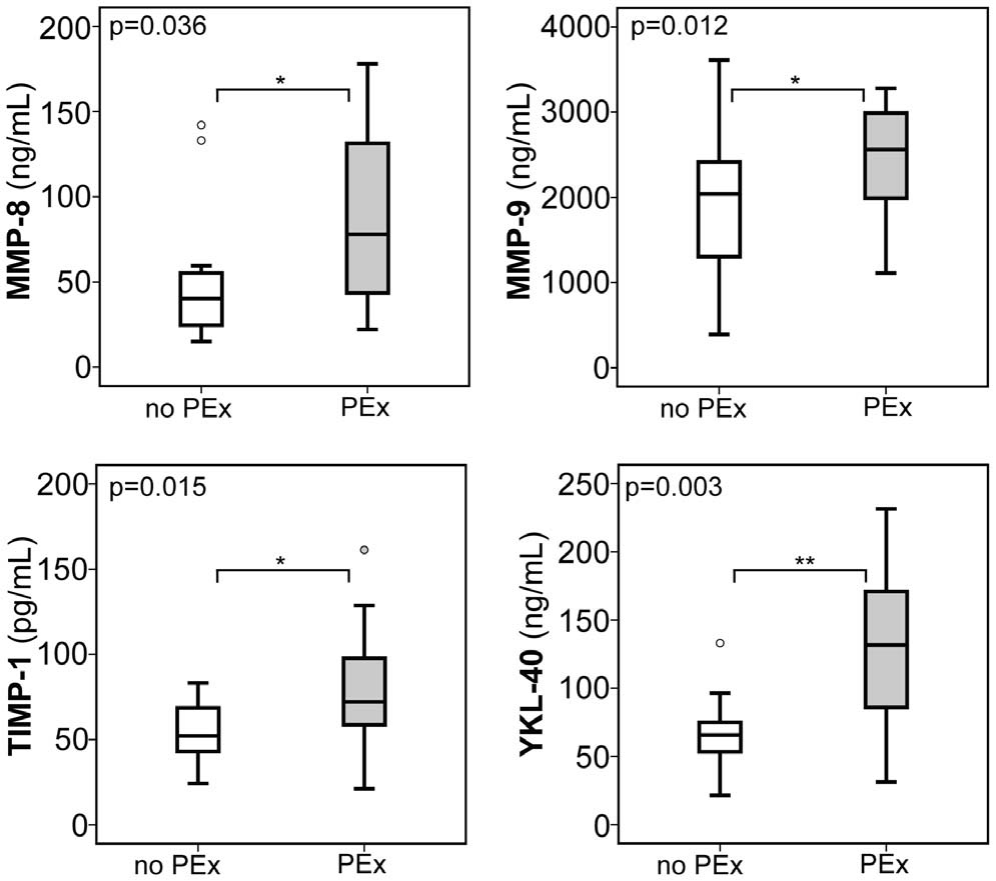
Serum expression of MMP-8, MMP-9, YKL-40, and TIMP-1 in adult CF patients with pulmonary exacerbation. Serum levels of YKL-40 were highly significant increased in adult CF patients suffering from pulmonary exacerbation (PEx) compared to those without PEx. Further, MMP-8, MMP-9, and TIMP-1 were significantly increased in serum from adults with PEx compared to CF patients without respiratory exacerbation. Upper and lower hinge: 75th and 25th percentile, respectively; Line: median value; Error bars: minimum and maximum. (*p<0.05, **p<0.01).

Finally, to further substantiate these associations of MMP-8, -9, YKL-40, and TIMP-1 expression with declined pulmonary function, we then hypothesized that CF patients with a pulmonary exacerbation (PEx) might also exhibit an increase of the aforementioned biomarkers. Of the 55 adult CF patients, 16 (29.1%) suffered from PEx and parameters of lung function were significantly impaired in patients suffering from PEx compared to CF patients without clinical signs of exacerbation. In these analyses, serum expression of YKL-40, MMP-8, MMP-9, and TIMP-1 were increased in CF patients with PEx compared to those without clinical signs of pulmonary exacerbation (**Fig. 5**). Importantly, all of the other biomarkers analyzed (MMP-1, MMP-2, MMP-13, TIMP-2, HA, PIIIP) were unaltered between CF patients with PEx and those without, thereby indicating that ECM markers are not generally up-regulated in CF patients with pulmonary exacerbation and declined lung function, but rather occurs selectively on the level of individual markers.

## Discussion

Progressively declining lung function in CF patients represents the most common cause of CF associated mortality [21], [22], [23]. Further, CF lung disease is characterized by increased bacterial endobronchial burden, repetitive cycles of non-resolving neutrophilic inflammation with impaired antibacterial killing and subsequent proteolytic destruction of pulmonary tissue [24]. Despite the central role that CF lung disease and recurrent episodes of pulmonary exacerbations play in clinical care of CF patients, surprisingly little non-invasive diagnostics apart from spirometry are available to adequately and routinely diagnose, monitor and follow-up CF lung disease. So far, about eighty experimental biomarkers, including inflammatory cytokines, acute phase reactants, and markers of oxidative stress, have been evaluated for the diagnosis of CF lung disease and pulmonary exacerbations [25]. Among these, especially neutrophil elastase within the sputum has been identified as a promising candidate marker that correlates with declined lung function and pulmonary inflammation in CF [26], [27], [28]. Further, it has been shown that neutrophil elastase exhibits profound immunologic effects within the lung such as activation of IL-8 [29], or cleavage of the matrix remodeling enzymes MMP-9 and TIMP-1 [30].

Based on this knowledge, we hypothesized that experimental matrix and neutrophil markers, determined within the serum, might act as novel non-invasive biomarkers of CF lung disease. As surrogate makers of CF lung disease, we used FEV1, VC and the FEV1/VC ratio, all of which are established indicators of CF lung disease and have been the primary outcome in many clinical trials. Using this approach, we found that serum expression of MMP-8, MMP-9, YKL-40, and TIMP-1 were significantly increased in adult and pediatric patients with moderate to severe CF lung disease compared to those without without a relevant decline in lung function or only mild CF lung disease. Importantly, expression of these markers was unaffected in the presence or absence of liver or pancreas disease, as shown in sub-analyses in this study. Thus, our results indicate that the aforementioned markers are rather specific for CF lung disease and hence might represent novel and non-invasive biomarkers of CF lung disease.

The family of Matrix Metalloproteinases (MMPs) consists of so far 28 identified proteolytic enzymes, which hold the potential to degrade virtually all components of the extracellular matrix and together with their specific inhibitors, the tissue inhibitors of metalloproteinases (TIMPs), are the main mediators of turnover and homeostasis of the ECM. Previous studies already have shown that deregulated MMP activity either leads to exaggerated ECM turnover, impaired repair, and scar formation or to ECM accumulation with subsequent tissue fibrosis in chronic lung diseases [31], [32], [33]. In addition to that, accumulating evidence suggests that MMPs indeed play a central role in the pathophysiology of lung tissue remodeling associated with CF. In this regard, several studies have documented enhanced MMP levels in BAL fluid or sputum of CF patients [34], [35], [36], [37] and especially MMP-9 has been shown to be associated with declined lung function and airway inflammation in CF [34], [35]. Further, basic research corroborates the etiologic role of MMP-8 and MMP-9 for pulmonary inflammation in CF [38], [39] and it was demonstrated that peroxisome proliferator-activated receptor (PPARγ) agonists can inhibit the inflammatory response in CF cell lines and CF mice at least in part by the attenuation of NF-κB-driven processes such as MMP-9 production [38]. Strikingly, Gaggar and co-workers found that MMP-8 and MMP-9 are involved in the production of a strong ECM-derived neutrophil chemoattractant, and therefore potentially contribute to neutrophil influx and airway damage in CF patients [39]. Within this study, we now show that both, MMP-8 and MMP-9 are increased in the serum of CF patients with CF lung disease and our results thus indicate that the previously observed increased MMP expression in bronchial tree of individuals with CF [34], [35], [36], [37] seems to be reflected in the blood compartment, revealing a potential role of MMPs as non-invasive and novel biomarkers of CF lung disease. Further, we observed robust and significantly increased serum levels of the chitinase-like protein YKL-40 in adults and children with CF lung disease. Importantly, a recent study was able to demonstrate that YKL-40 levels are increased in sputum and serum of CF patients compared to healthy control individuals [40], and in both CF patients and bENaC-Tg mice, a murine model mirroring CF lung disease [41], airway levels of YKL-40 and its murine analogue BRP-39 airway levels correlated with the severity of pulmonary obstruction [40]. Further, two YKL-40 SNPs were found to modulate age-adjusted lung function in CF patients [40]. Besides its role in CF, it also has been shown that YKL-40 serum levels are increased in patients with severe asthma [42] and chronic obstructive pulmonary disease (COPD) [43], both of which are also characterized by neutrophilic inflammation. Together with these data, our findings suggest that YKL-40 might indeed represent a novel biomarker of CF lung disease.

Importantly, in order to further substantiate our findings of increased MMP-8, MMP-9, and YKL-40 expression in CF lung disease, we analyzed their expression in adult CF patients currently suffering from PEx. In these analyses, we found that PEx patients exhibit even higher levels of MMP-8, MMP-9, and YKL-40 compared to CF patients without clinical signs of pulmonary exacerbation. These findings have important implications. Firstly, until now neither a standardized definition of PEx has been published nor accepted and as a result the presence of an exacerbation remains essentially a clinical diagnosis [16], [44]. Thus, routine markers reliably indicating the occurrence of pulmonary exacerbations in CF are of clinical need, and our results indicate that MMP-8, MMP-9, and YKL-40 might indeed be utilized as novel serum markers facilitating the diagnosis of PEx. Secondly, from the results of the current study, the question about the cell types responsible for the increased MMP and YKL-40 production arises, and although we were not able to allocate the MMP and YKL-40 secretion to a certain cell type, it can be speculated that the observed increases in PEx reflect the extent of neutrophilic airway inflammation. Although this aspect clearly is speculative in its nature, this view is supported by the notion that neutrophils represent a main source of MMP-production, especially of MMP-8 ( = neutrophilic collagenase) and MMP-9, and have also been shown to store YKL-40 within their secretory granula [24]. However, to further substantiate this hypothesis, further studies are needed in which biomarker levels in serum and sputum are assessed simultaneously along with identification of the main cellular sources of these on serum, sputum, and tissue level.

Possible limitations of this study should also be addressed, one of which is the impossibility to directly relate the increased serological biomarker expression to pulmonary tissue as the origin of these biomarkers. Especially members of the MMP family and YKL-40 have also been shown to be associated with other systemic diseases such as cardiovascular disease or diabetes [45], [46], [47], [48], [49]. On the other hand, as already discussed above, members of the MMP family and YKL-40 have been shown to be increased in the airways of CF patients [10], [34], [35], [36], [37], [40], [50] and thus, it might well be speculated that the observed increased serum levels are indeed derived from the bronchial tree of CF patients. As a further limitation, the number of patients without pancreatic insufficiency was rather small. This problem results from the natural course of disease, with 80 to 90% of all CF patients being pancreatic insufficient [51], [52] and an onset of pancreatic insufficiency in very early stages of life [51], thus making it challenging to recruit large number of CF patients without pancreatic insufficiency. Hence, although the subgroup analysis of biomarker expression with regard to the presence or absence of pancreatic insufficiency presented in this study is exploratory in its nature, the results do support the concept that the up-regulation of individual biomarkers in the serum of CF patients is associated with declined pulmonary function, but not with the presence or absence of CFLD or PI.

In summary, we have shown that adult and pediatric CF patients with moderate to severe CF lung disease exhibit increased serum levels of MMP-8, MMP-9, and YKL-40 and that this association occurs rather organ specific as is not influenced by the co-existence or absence of CFLD. Further, CF patients suffering from pulmonary exacerbation exhibit further increased serum levels of these biomarkers. Hence, MMP-8, MMP-9, and YKL-40 might represent novel serological markers of CF lung disease and pulmonary exacerbations.

## Supporting Information

S1 Table **Serum expression of experimental matrix and neutrophil markers in adult CF patients according to the relative forced expiratory volume in one second (FEV1).**
(DOCX)

S2 Table **Serum expression of matrix and neutrophil markers in adult CF patients according to the relative vital capacity (VC).**
(DOCX)

S3 Table **Serum expression of matrix and neutrophil markers in adult CF patients according to the ratio FEV1/VC.**
(DOCX)

S4 Table **Serum expression of matrix and neutrophil markers in pediatric CF patients according to the relative forced expiratory volume in one second (FEV1).**
(DOCX)

S5 Table **Serum expression of matrix and neutrophil markers in pediatric CF patients according to the relative vital capacity (VC).**
(DOCX)

S6 Table **Serum expression of matrix and neutrophil markers in pediatric CF patients according to the ratio FEV1/VC.**
(DOCX)
