# Supplementary material for: Identification of Neutrophil Activation Markers as Novel Surrogate Markers of CF Lung Disease
Source: PLoS One. 2014 Dec 29;9(12):e115847. doi: 10.1371/journal.pone.0115847 (PMC4278831; doi:10.1371/journal.pone.0115847)
Supplement: S1 Table — Serum expression of experimental matrix and neutrophil markers in adult CF patients according to the relative forced expiratory volume in one second (FEV1). (DOCX) [file pone.0115847.s001.docx]

|  | **FEV1≥80%** | **FEV1<80%** | ***Significance*** |
| --- | --- | --- | --- |
| **MMP-1** (ng/mL)  Mean ± SD  Median (range) | 1157 ± 420  1295 (510 – 1790) | 1562 ± 865  1285 (410 – 4160) | p=0.369 |
| **MMP-2** (ng/mL)  Mean ± SD  Median (range) | 13.4 ± 2.9  14.1 (6.7 – 17) | 12.8 ± 2.3  12.9 (8.9 – 18.6) | p=0.244 |
| **MMP-13** (ng/mL)  Mean ± SD  Median (range) | 21.1 ± 18.1  15.5 (1.6 – 44.6) | 44.3 ± 140  4.96 (0 – 651) | p=0.374 |
| **TIMP-2** (pg/mL)  Mean ± SD  Median (range) | 118.5 ± 25.3  117.6 (80.8 – 157) | 130.2 ± 25.6  123.1 (85.3 – 211.1) | p=0.247 |
| **HA** (ng/mL)  Mean ± SD  Median (range) | 26.2 ± 26.1  21.2 (3.6 – 92) | 29.3 ± 21.4  29 (0.8 – 127.5) | p=0.275 |
| **PIIIP** (ng/mL)  Mean ± SD  Median (range) | 11.7 ± 15.4  4.6 (0 – 45.7) | 15.3 ± 27.7  4.9 (1.2 – 136.1) | p=0.721 |

**Table S1:** Serum expression of experimental matrix and neutrophil markers in adult CF patients according to the relative forced expiratory volume in one second (FEV1).
