# Supplementary material for: Identification of Neutrophil Activation Markers as Novel Surrogate Markers of CF Lung Disease
Source: PLoS One. 2014 Dec 29;9(12):e115847. doi: 10.1371/journal.pone.0115847 (PMC4278831; doi:10.1371/journal.pone.0115847)
Supplement: S2 Table — Serum expression of matrix and neutrophil markers in adult CF patients according to the relative vital capacity (VC). (DOCX) [file pone.0115847.s002.docx]

|  | **VC≥80%** | **VC<80%** | ***Significance*** |
| --- | --- | --- | --- |
| **MMP-1** (ng/mL)  Mean ± SD  Median (range) | 1338 ± 661  1280 (410 – 3430) | 1649 ± 941  1310 (410 – 4160) | p=0.345 |
| **MMP-2** (ng/mL)  Mean ± SD  Median (range) | 13.1 ± 2.8  13.2 (6.7 – 18.6) | 12.8 ± 1.8  12.8 (9.7 – 16.5) | p=0.566 |
| **MMP-13** (ng/mL)  Mean ± SD  Median (range) | 69.3 ± 176  22.7 (1.6 – 651.8) | 12.4 ± 13.5  5 (0 – 41) | p=0.184 |
| **TIMP-2** (pg/mL)  Mean ± SD  Median (range) | 126 ± 29.2  119.1 (80.8 – 211.1) | 130.1 ± 21.7  124.1 (85.3 – 179.1) | p=0.406 |
| **HA** (ng/mL)  Mean ± SD  Median (range) | 32.8 ± 26.8  28.1 (3.6 – 127.5) | 24.1 ± 14.6  23 (0.8 – 53.6) | p=0.378 |
| **PIIIP** (ng/mL)  Mean ± SD  Median (range) | 19.2 ± 34  5.3 (0 – 136.1) | 10.1 ± 27.7  4.9 (1.6 – 50.5) | p=0.674 |

**Table S2**: Serum expression of matrix and neutrophil markers in adult CF patients according to the relative vital capacity (VC).
