# Supplementary material for: Identification of Neutrophil Activation Markers as Novel Surrogate Markers of CF Lung Disease
Source: PLoS One. 2014 Dec 29;9(12):e115847. doi: 10.1371/journal.pone.0115847 (PMC4278831; doi:10.1371/journal.pone.0115847)
Supplement: S3 Table — Serum expression of matrix and neutrophil markers in adult CF patients according to the ratio FEV1/VC. (DOCX) [file pone.0115847.s003.docx]

|  | **FEV1/VC≥70%** | **FEV1/VC<70%** | ***Significance*** |
| --- | --- | --- | --- |
| **MMP-1** (ng/mL)  Mean ± SD  Median (range) | 1464 ± 726  1310 (510 – 3160) | 1490 ± 860  1270 (416 – 4160) | p=0.751 |
| **MMP-2** (ng/mL)  Mean ± SD  Median (range) | 12.4 ± 2.8  12.4 (6.6 – 17.1) | 13.1 ± 2.2  13.2 (9 – 18.6) | p=0.442 |
| **MMP-13** (ng/mL)  Mean ± SD  Median (range) | 15.2 ± 15.4  8.4 (1.6 – 44.6) | 51.7 ± 150.7  8.4 (0 – 652) | p=0.982 |
| **TIMP-2** (pg/mL)  Mean ± SD  Median (range) | 118.7 ± 26  108.7 (80.8 – 160.1) | 132 ± 24.8  124.4 (85.3 – 211.1) | p=0.075 |
| **HA** (ng/mL)  Mean ± SD  Median (range) | 25.9 ± 24.8  24.8 (3.6 – 92) | 29.9 ± 23  28.7 (0.8 – 127.6) | p=0.416 |
| **PIIIP** (ng/mL)  Mean ± SD  Median (range) | 16.2 ± 33.8  4.3 (0 – 136.1) | 14 ± 22.1  5.1 (1.2 – 122.1) | p=0.539 |

**Table S3:** Serum expression of matrix and neutrophil markers in adult CF patients according to the ratio FEV1/VC.
