# Supplementary material for: Identification of Neutrophil Activation Markers as Novel Surrogate Markers of CF Lung Disease
Source: PLoS One. 2014 Dec 29;9(12):e115847. doi: 10.1371/journal.pone.0115847 (PMC4278831; doi:10.1371/journal.pone.0115847)
Supplement: S4 Table — Serum expression of matrix and neutrophil markers in pediatric CF patients according to the relative forced expiratory volume in one second (FEV1). (DOCX) [file pone.0115847.s004.docx]

|  | **FEV1≥80%** | **FEV<80%** | ***Significance*** |
| --- | --- | --- | --- |
| **MMP-1** (ng/mL)  Mean ± SD  Median (range) | 1163 ± 816  810 (240 – 3460) | 1493 ± 1114  985 (350 – 3430) | p=0.628 |
| **MMP-2** (ng/mL)  Mean ± SD  Median (range) | 19.7 ± 4.5  19.4 (12.6 – 28.3) | 16.2 ± 3.3  15 (13.3 – 23.1) | p=0.057 |
| **MMP-13** (ng/mL)  Mean ± SD  Median (range) | 82.1 ± 56.9  70.8 (22.7 – 248.6) | 48.2 ± 28.3  44.7 (11.9 – 84) | p=0.157 |
| **TIMP-2** (pg/mL)  Mean ± SD  Median (range) | 138.7 ± 21.4  137.3 (94.7 – 170.7) | 139.2 ± 35.2  137.5 (100.4 – 210) | p=0.842 |
| **HA** (ng/mL)  Mean ± SD  Median (range) | 20.4 ± 12.5  22.3 (0 – 42.8) | 21.3 ± 14.8  15.7 (3.4 – 45.1) | p=0.932 |
| **PIIIP** (ng/mL)  Mean ± SD  Median (range) | 6.7 ± 7.5  2.9 (0 – 24.9) | 8.8 ± 7.6  5 (3.2 – 22.9) | p=0.153 |

**Table S4:** Serum expression of matrix and neutrophil markers in pediatric CF patients according to the relative forced expiratory volume in one second (FEV1).
