# Supplementary material for: Identification of Neutrophil Activation Markers as Novel Surrogate Markers of CF Lung Disease
Source: PLoS One. 2014 Dec 29;9(12):e115847. doi: 10.1371/journal.pone.0115847 (PMC4278831; doi:10.1371/journal.pone.0115847)
Supplement: S5 Table — Serum expression of matrix and neutrophil markers in pediatric CF patients according to the relative vital capacity (VC). (DOCX) [file pone.0115847.s005.docx]

|  | **VC≥80%** | **VC<80%** | ***Significance*** |
| --- | --- | --- | --- |
| **MMP-1** (ng/mL)  Mean ± SD  Median (range) | 1150 ± 790  805 (240 – 3460) | 1463 ± 1141  990 (350 – 3430) | p=0.683 |
| **MMP-2** (ng/mL)  Mean ± SD  Median (range) | 19.2 ± 4.9  19.4 (12.6 – 28.3) | 16.6 ± 2.2  16.3 (13.3 – 19.4) | p=0.238 |
| **MMP-13** (ng/mL)  Mean ± SD  Median (range) | 73.8 ± 56.2  64.2 (0 – 248.6) | 57.5 ± 43.9  43.7 (11.9 – 139.4) | p=0.495 |
| **TIMP-2** (pg/mL)  Mean ± SD  Median (range) | 140.4 ± 29  143.6 (94.7 – 210) | 131 ± 18.1  134.5 (100.4 – 158.2) | p=0.531 |
| **HA** (ng/mL)  Mean ± SD  Median (range) | 21.6 ± 13.2  23.3 (0 – 42.8) | 18.3 ± 12.2  13.2 (9.2 – 45.1) | p=0.429 |
| **PIIIP** (ng/mL)  Mean ± SD  Median (range) | 13.2 ± 31.3  3.2 (0 – 136.1) | 10.3 ± 8.6  5.9 (3.6 – 24.9) | p=0.090 |

**Table S5:** Serum expression of matrix and neutrophil markers in pediatric CF patients according to the relative vital capacity (VC).
