# Supplementary material for: Identification of Neutrophil Activation Markers as Novel Surrogate Markers of CF Lung Disease
Source: PLoS One. 2014 Dec 29;9(12):e115847. doi: 10.1371/journal.pone.0115847 (PMC4278831; doi:10.1371/journal.pone.0115847)
Supplement: S6 Table — Serum expression of matrix and neutrophil markers in pediatric CF patients according to the ratio FEV1/VC. (DOCX) [file pone.0115847.s006.docx]

|  | **FEV1/VC≥70%** | **FEV1/VC<70%** | ***Significance*** |
| --- | --- | --- | --- |
| **MMP-1** (ng/mL)  Mean ± SD  Median (range) | 1095 ± 798  800 (240 – 3460) | 1657 ± 1094  1170 (640 – 3430) | p=0.188 |
| **MMP-2** (ng/mL)  Mean ± SD  Median (range) | 19.2 ± 4.7  19 (12.6 – 28.3) | 16.3 ± 2.7  16.4 (13.3 – 20.7) | p=0.188 |
| **MMP-13** (ng/mL)  Mean ± SD  Median (range) | 80.3 ± 56.2  72.7 (0 – 248.6) | 37.7 ± 21.1  37.3 (11.9 – 76.4) | p=0.035 |
| **TIMP-2** (pg/mL)  Mean ± SD  Median (range) | 140.3 ± 26.1  137.3 (94.7 – 210) | 130.2 ± 26.8  120 (100.4 – 170.7) | p=0.572 |
| **HA** (ng/mL)  Mean ± SD  Median (range) | 18 ± 12.5  15 (0 – 42.8) | 27.6 ± 11.5  26.8 (13.4 – 45.1) | p=0.073 |
| **PIIIP** (ng/mL)  Mean ± SD  Median (range) | 13.4 ± 30.4  4.2 (0 – 136.1) | 9.3 ± 9.1  4.7 (2.5 – 22.9) | p=0.651 |

**Table S6:** Serum expression of matrix and neutrophil markers in pediatric CF patients according to the ratio FEV1/VC.
